# State of the Art in the Role of Endovascular Embolization in the Management of Brain Arteriovenous Malformations—A Systematic Review

**DOI:** 10.3390/jcm11237208

**Published:** 2022-12-04

**Authors:** Miłosz Pinkiewicz, Mateusz Pinkiewicz, Jerzy Walecki, Michał Zawadzki

**Affiliations:** 1 Faculty of Medicine, Wroclaw Medical University, 50-367 Wrocław, Poland; 2 Department of Diagnostic Imaging, Mazowiecki Regional Hospital in Siedlce, 08-110 Siedlce, Poland; 3 Department of Radiology, the Central Clinical Hospital of the Ministry of Interior and Administration, Centre of Postgraduate Medical Education, 02-507 Warsaw, Poland

**Keywords:** brain arteriovenous malformations, endovascular embolization, preradiosurgical embolization, presurgical embolization, transvenous approach, transarterial approach

## Abstract

As a significant cause of intracerebral hemorrhages, seizures, and neurological decline, brain arteriovenous malformations (bAVMs) are a rare group of complex vascular lesions with devastating implications for patients’ quality of life. Although the concerted effort of the scientific community has improved our understanding of bAVM biology, the exact mechanism continues to be elucidated. Furthermore, to this day, due to the high heterogeneity of bAVMs as well as the lack of objective data brought by the lack of evaluative and comparative studies, there is no clear consensus on the treatment of this life-threatening and dynamic disease. As a consequence, patients often fall short of obtaining the optimal treatment. Endovascular embolization is an inherent part of multidisciplinary bAVM management that can be used in various clinical scenarios, each with different objectives. Well-trained neuro-interventional centers are proficient at curing bAVMs that are smaller than 3 cm; are located superficially in noneloquent areas; and have fewer, larger, and less tortuous feeding arteries. The transvenous approach is an emerging effective and safe technique that potentially offers a chance to cure previously untreatable bAVMs. This review provides the state of the art in all aspects of endovascular embolization in the management of bAVMs.

## 1. Introduction

Arteriovenous malformations of the brain involve a dense tangle of anomalous connections between dilated cerebral arteries and veins located within the brain parenchyma [1,2]. These lesions are known to form at the interface between the arterial and venous endothelium [2]. The abnormal vascular organization at the subarterial level of these vessels and the lack of a capillary bed lead to a high-flow, low-resistance arteriovenous shunt [1,2]. The resulting blood-flow conditions significantly increase the risk of intracerebral hemorrhage and seizures, with the former being the most common manifestation as well as the primary source of morbidity and mortality due to bAVMs, especially in children and young adults [3,4]. Early hemorrhage symptoms are often mild, with bleeding typically limited to the brain arteriovenous malformation itself or stemming from the venous side of the malformation [5,6,7]. Aside from these most common manifestations, patients may develop focal neurologic deficits and headaches independently of cerebral bleeding [1,2]. Generally, ruptured bAVMs manifest in intraparenchymal, subarachnoid, and intraventricular hemorrhages. However, there have been reported cases, although only five, of acute spontaneous subdural hematoma (ASSDH) following a bAVM rupture [8]. Acute blood in the adjacent parenchyma, ventricles, or subarachnoid space can be seen on computed tomography (CT) scans and magnetic resonance imaging (MRI). Although the rupture of a bAVM is usually associated with less severe clinical consequences than intracranial hemorrhage due to other causes, it can be fatal or severely disabling. Similar to aneurysms, bAVM ruptures can be preceded by a minor leak or “sentinel hemorrhage”. However, in contrast to aneurysms, such bAVM microhemorrhages do not have any clinical manifestations, often causing them to be undetected. Although the bleeding can expand beyond the lesion into the surrounding parenchyma, it is generally located intralesionally. With time, hemosiderin is deposited in the adjacent tissue, which induces inflammation, gliosis, and scarring. These alterations often provoke seizures. Given that CT scanning has limited sensitivity to subacute or remote hemorrhage, using MRI, particularly with iron-sensitive imaging sequences such as gradient echo T2* and more advanced techniques for susceptibility-sensitive imaging such as susceptibility-weighted imaging, is highly sensitive in detecting bAVM microhemorrhages.

Numerous reports of bAVMs acquired de novo in patients several years after cerebral injury, the lack of parenchymal bAVMs on prenatal ultrasound, active inflammatory and angiogenic processes, and recurrences after surgical treatment and remodeling observed during follow-up have prompted the research community to strongly question the static, congenital nature of this pathological entity [3,9,10,11]. As a consequence, the paradigm has shifted toward accepting that bAVMs can also form postnatally. The interplay between hemodynamic and flow-related phenomena, as well as vasculogenesis, are thought to be responsible for the dynamic nature of bAVMs [12].

The contemporary management of these lesions, which can differ in size, location, morphology, and angioarchitecture, aims to obliterate the nidus or provide a complete endoluminal closure [4,9]. Observation, surgical resection, embolization, stereotactic radiosurgery (SRS), or multimodality treatment strategies comprise the current therapeutic arsenal for treating bAVMs [4,9]. To this day, due to the limited understanding of the biology of bAVMs, pharmacological interventions rely solely on the nonspecific control of symptomatology such as headache and seizures because there is no specific medical therapy available to directly treat bAVMs or decrease the spontaneous rupture risk [4]. It is still a matter of considerable debate whether preventive lesion eradication offers a clinical benefit for patients diagnosed with an unruptured brain arteriovenous malformation [7]. The lack of reporting terminology based on current practice and imaging technology that could provide a frame of reference, as well as the scarcity of adequate animal models, have significantly impeded research progress and new therapy development [13]. However, the ongoing clinical trials that are relying on uniform reporting standards aim to catch up and establish clear guidelines regarding the most effective bAVM management [4,8].

As much as this review aimed to provide a comprehensive, albeit concise, review of the epidemiology, pathobiology, genetic factors, and risk of cerebral hemorrhage, the prevailing part of this review focuses on endovascular embolization in the management of bAVMs.

## 2. Methodological Approach

### 2.1. Search Strategy and Selection Criteria

A systematic literature review was carried out to review all available relevant data. During the article-selection process, the authors followed the recommendations made in the Preferred Reporting Items for Systematic Reviews and Meta-Analyses (PRISMA). All authors independently searched the Medline/PubMed, Cochrane, Google Scholar, Scielo, and PEDro databases using the following keywords “arterio-venous malformation”, “brain arteriovenous malformation”, “presurgical embolisation “preradiosurgical embolisation”, “radiosurgery”, “microsurgical resection”, “endovascular embolisation”, “transvenous approach”. An additional search included the Scielo and PEDro databases. The last search was conducted in August 2022. The references of the publications of interest were also screened for relevant papers.

### 2.2. Study Selection and Data Extraction

The full text of all of the selected articles were read. Only papers written in English were considered. Non-peer-reviewed papers and records not available in the full text were not included. In addition, studies were excluded if they had incomplete or missing data. We also excluded conference abstracts. The eligibility and quality of publications were independently evaluated by three reviewers. We chose articles for inclusion on the grounds of the study quality and design. The primary selection had no limitations regarding the publication date. We included studies concerning the epidemiology of bAVMs as well as the pathobiology, genetic factors, complications, and consequences of untreated bAVMs. With regard to treatment, we focused solely on the role of endovascular embolization in the management of bAVMS while illuminating the technical aspects as well as categorizing the role of endovascular embolization into three categories: presurgical and preradiosurgical endovascular embolization in the acute settings, and curative. Additionally, we reviewed studies that concerned the use of the transvenous approach. Last but not least, we included clinical trials that concerned the use of the endovascular approach in the management of bAVMs.

The judgments concerning the risk of bias were formed by a single reviewer and subsequently double-checked by another reviewer

## 3. Results

**Figure 1:**
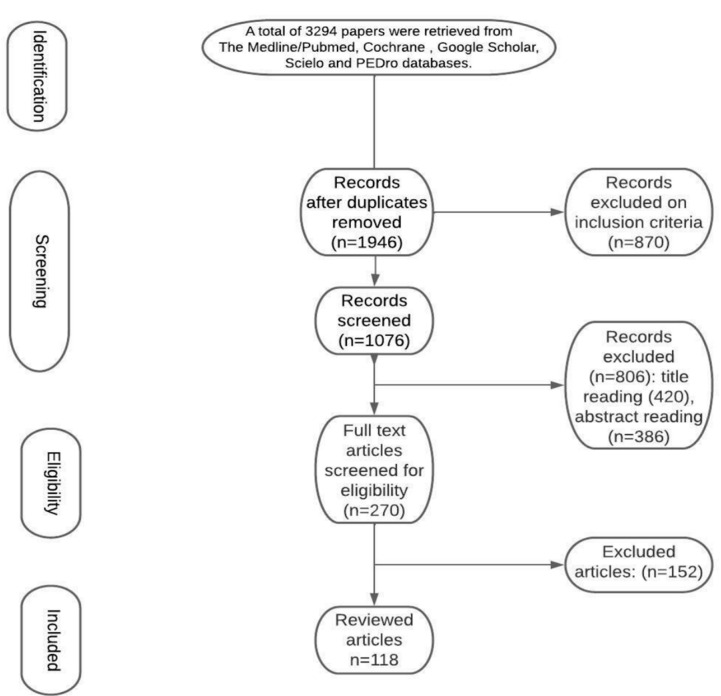
Flow diagram representing our process for article selection.

A total of 3294 papers were retrieved from the Medline/PubMed, Cochrane, Google Scholar, Scielo, and PEDro databases. Screening for duplicates and their removal resulted in a total of 1946 articles. Subsequently, we excluded 870 articles due to the language and study design. The titles or abstracts of 1076 articles were screened, which obtained 270 papers that did not meet any of the exclusion criterion. After full-text evaluation, we excluded 152 papers. This led to the inclusion of 118 articles. Figure 1 demonstrates our process for article selection.

### 3.1. Epidemiology

Hitherto, given that 88% of patients are asymptomatic, establishing a true prevalence rate is extremely complicated if not even impossible [14]. However, due to the widespread use of advanced imaging modalities, there has been an increase in the incidental discoveries of bAVM [1,2]. Brain arteriovenous malformations have an incidence rate of 0.89–2.05 per 100,000 person-years in Western societies [15,16,17,18]. According to a systematic review, the incidence of bAVMs is approximately 1 per 100,000 per year in unselected populations, with the point prevalence in adults being equal to 18 cases per 100,000 [16]. It has been estimated that bAVMs account for 1–2% of all strokes, 3% of strokes in young adults, 9% of subarachnoid hemorrhages, and 4% of all primary intracranial hemorrhages [15,19]. These bAVMs are responsible for 25% of hemorrhagic strokes in adults below the age of 50 years [14]. Approximately 15% of people suffering from bAVMs are asymptomatic at the time of detection, whereas around 20% demonstrate seizures, and about two-thirds present an intracranial hemorrhage [19]. The reported mortality rates in bAVM patients range from 0.7 to 2.9% per year [20]. Observational studies reported that the mortality rate after intracranial hemorrhage from bAVMs rupture ranged from 12 to 66.7% [6,21,22,23]. According to a systematic review of 18 studies with a total of 8418 bAVMs, the average annualized hemorrhage rate was 2.2% for unruptured bAVMs and 4.3% for bAVMs that presented with bleeding [24].

### 3.2. Biology

Brain arteriovenous malformations are anatomically complex entities [25]. Given that blood flow around the malformation prefers the low-resistance shunt through the malformation rather than the surrounding vessels, bAVMs can result in a steal phenomenon, with the degree of vascular steal being inversely proportional to the resistance within the bAVM itself [12,25,26]. Eventually, the resulting perilesional hypoperfusion can cause the dilation of the perinidal capillary network and the involvement of leptomeningeal collaterals [12,26]. In consequence, niduses are surrounded by a small perinidal dilated capillary network that is connected not only to the nidus, feeding arteries, and draining veins via arterioles and venules but also to normal capillaries, arterioles, and venules [25,27]. Brain arteriovenous malformations can have single or multiple compartments. Monocompartmental lesions have a single compact nidus of one feeder and one or more draining veins; the elimination of the single feeder results in the collapse of the malformation [12]. However, in cases of multicompartmental bAVMs, several feeders and draining veins can be divided into compartments that are connected or separated by small nonfunctional or even functional brain parenchyma [12]. The term “hidden compartments” is used to define unfilled compartments on angiography, which cause bAVMs to appear to grow by serial filling of small or large hidden compartments over time or after therapy [12].

Although the concerted effort of the scientific community has improved our understanding of bAVM pathology, the exact mechanism continues to be elucidated [3,15]. As much as brain arteriovenous malformations are common in patients suffering from hereditary hemorrhagic telangiectasia and capillary malformation–arteriovenous malformation syndrome (CM-AVM), approximately 95% of bAVMs are sporadic [28]. The two-hit model has been hypothesized to underlie the sporadic arteriovenous malformations of the brain; as an insult to the brain, it acts as a “second-hit” to an existing genetic aberrancy [3,9,10,11]. Hypoxic tissues, unlike oxygenated tissues, have an increased expression of HIF-1alpha, which in turn promotes VEGF and VEGFR expression. For this reason, the overexpression of HIF-1alpha in human bAVMS led researchers to suggest the potential role of a hypoxic incident in the pathogenesis of bAVMs [29]. Likewise, intracranial venous hypertension has been hypothesized as a causative agent of bAVMS because it also induces the expression of HIF-1 and therefore stimulates the expression of VEGF [4,9,30]. Molecular and histological examinations of human bAVM specimens demonstrated inflammatory cell infiltrations, increased levels of angiogenic factors, and inflammatory cells [4,31,32,33,34,35,36,37,38,39,40,41,42]. Inflammation and extracellular-matrix remodeling are thought to play a role in bAVM growth and rupture because neutrophilia and increased macrophage migration inhibitory factor could potentially provoke the instability of nidal vasculature [4,9,10,11]. Studies have associated metalloproteinases with bAVM growth and rupture because by degrading pericellular substances, these proteolytic enzymes led to vascular destabilization and altered angiogenesis [9,43]. Recent studies highlighted that the brain pericyte number and coverage are decreased in sporadic bAVMs and are lowest in patients with a prior rupture. In unruptured bAVMs, a reduced number of pericytes correlated with the severity of microhemorrhages and a faster rate of blood flow through the bAVM nidus [44]. According to Winkler et al., these findings suggested that pericytes are associated with and may explain vascular fragility and hemodynamic changes in bAVMs [44].

It is well established that immoderate angiogenesis and vascular remodeling have a significant role in the formation and progression of cerebral AVMs [1,2,3,4,9,26,31]. Nevertheless, an extensive body of literature lent credence to the notion that dysregulation of angiogenesis by vascular endothelial growth factor may be the cause of recurrences following angiographic cure after microsurgery or embolization [11]. This problem is especially pronounced in young patients; a recent meta-analysis showed that bAVM recurrence occurred in 2.7% of reported adult series and in nearly 10%–15% of children [11]. This is attributable to the fact that children have higher vascular endothelial growth factor levels, stronger vascular endothelial growth factor receptor expression, and faster endothelial cell turnover [10,11].

### 3.3. Genetic Factors

Although bAVMs have a higher incidence in patients suffering from hereditary hemorrhagic telangiectasia, the endoglin expression observed in sporadic bAVMs was decreased in lesions obtained from patients with hereditary hemorrhagic telangiectasia, thus failing to prove a positive association between endoglin and the incidence of sporadic bAVMs [37,38,39,40]. In light of these findings, Oka et al. suggested that rather than a single pathway, it is much more likely that multiple or heterogeneous pathways are involved in the pathobiology of bAVMs [42].

Somatic activating *KRAS* or *BRAF* mutations are present in approximately 80% of patients with sporadic bAVMs [45,46,47,48,49]. A 2018 study found somatic activating *KRAS* mutations in tissue samples from 45 of the 72 patients with bAVMs, with the majority constituted by activating mutations (G12D and G12V) [45]. Consequently, researchers hypothesized that KRAS-induced activation of the MAPK–ERK signaling pathway in brain endothelial cells that leads to the expression of angiogenesis-related genes and enhanced migratory behavior plays a role in bAVM pathology [42,45]. Studies using postnatal and adult mice as well as embryonic zebrafish demonstrated that active KRAS expression in the endothelium was enough to induce bAVMs [50]. Recently, in addition to confirming that KRAS mutations (G12V and G12D) are the most prevalent somatic mutation in bAVM tissue, Gao et al. reported that PDGFRB and CRKL genes with candidate somatic mutations were detected more often than expected [51]. A recent study demonstrated a significant association between the angiopoietin-like protein 8 regulatory pathway and bAVMs [52]. Likewise, *SLC19A3* is a disease-associated gene of bAVMs and has a potential domain-specific effect [52]. Future studies should focus on evaluating the role of these mutations in the pathobiology of bAVMs.

### 3.4. The Risk of Cerebral Hemorrhages and Other Complications

Although the annual risk of hemorrhages due to bAVMs is around 3% depending on the clinical and anatomical factors, the risk can range from 1% to 33% [1]. Identifying risk factors for hemorrhage is essential in selecting patients who would benefit the most from the treatment; however, small sample sizes or selection biases of cases have stood in the way [14]. The most important factors that significantly increase the likelihood of bAVM rupture include a history of hemorrhage, high blood pressure, and intranidal aneurysm [1,4,9,32]. According to Solomon et al., the risk of cerebral bleeding was also higher in cases of deeply located malformations within the brain or brain stem, eloquent cortical location, or when there was a berry aneurysm on the feeding artery of a bAVM [1,53,54]. Numerous studies have shown that impairment of venous drainage of an AVM was associated with a higher risk of hemorrhage [1,4,9,30,54]. Venous outflow can be compromised by deep venous drainage, the presence of a single draining vein, or in the case of multiple draining veins, venous stenosis, which is known to occur most commonly near the dural venous sinus junction. In contrast, arterial feeder stenosis has a protective effect with a decreased probability of intracranial hemorrhage [30]. However, as much as the association between the increased risk of hemorrhage and AVM-induced venous stenosis is well established, many natural history studies do not describe the incidence of venous stenosis [30,33,53].

Following a recent systematic review, cytokines (IL-6, IL-17A, IL-1β, and TNF-α), NOTCH pathways, MMP-9, and VEGFA were associated with a higher hemorrhage risk in patients with bAVM [6].

There have been reports of blood–brain barrier (BBB) disruption and resulting microhemorrhages in patients with unruptured bAVMs. Such manifestations could predict future ruptures [2]. Following a multicenter study, around 25% of survivors of cerebral bleeding had no neurologic deficit, 30% manifested with a mild-to-moderate deficit, and 45% suffered from a severe deficit. Three months after the hemorrhage, approximately 20% of those patients died and one-third continued to manifest moderate disability [1,55]. Considering the high rate of serious disability after hemorrhage (23–85%) and the fact that unruptured bAVMs can bleed in the future, bAVMs constitute a significant clinical problem associated with grave risks [14,15]. Brain micro-arteriovenous malformations, which are defined as bAVMs that are just visible on angiography with a nidus size between 0.5 and 1 cm, are often deeply located in the brain and have inconspicuous feeding arteries and single draining veins [56]. Micro-bAVMs, which most typically manifest with an intracerebral hematoma (ICH), can lead to large hemorrhages and are associated with significant neurological deficits [57].

Aside from intracranial hemorrhages, venous congestion that results from the stenosis of the draining veins, perinidal gliogenesis, alteration of the blood–brain barrier, hypertension attributable to high-flow shunts, or an arterial steal phenomenon may provoke neurological decline and epilepsy [9,58,59,60]. After a hemorrhage, epilepsy is the second most common revealing condition and affects approximately a quarter of patients after a bAVM rupture [60]. A BAVM rupture is the most important risk factor for epilepsy in this group of patients; however, a cortical or temporal lobe location of the nidus, a size of the nidus >3 cm, superficial venous drainage, or a supply by the middle cerebral artery have also been associated with a higher risk of epilepsy [60]. Although the exact mechanism of epileptogenesis in bAVMs continues to be unknown, potential mechanisms involve perinidal gliogenesis, alteration of the blood–brain barrier, and ischemia of the adjacent parenchyma via a stealing of the cerebral blood flow [9,58,60]. Given the high incidence of seizures in bAVM patients, it is crucial to evaluate different treatment modalities and seizure outcomes with clinical trials that employ a standardized seizure scale (Engel scale) [9,58,60].

Accounting for 5% to 20% of bAVMs, occipital lobe arteriovenous malformations are associated with a high risk of visual impairment due to their intimate relation with the visual anatomical structures [61,62,63,64]. As much as 37% to 51% of patients with an occipital bAVM demonstrate visual impairment with lesions in the primary visual cortex that lead to a loss of conscious access to the majority of the visual information in the contralateral visual field (VF) [61,62,63,64]. The nidus size, involvement of the calcarine artery, the occipital gyrus O5–O6 location, and deep venous drainage have been identified as risk factors for the deterioration of visual function [62]. Although there have been only a few studies that reported visual outcomes in patients with occipital bAVMs after an endovascular embolization, Smajda et al. reported post-treatment worsening of the VF in 24 of the treated patients (30%); 3 patients (9%) had ruptured bAVMs and 21 patients (46%) had unruptured bAVMs [61]. Similarly, Yang et al. reported visual disturbances in 3/8 patients who underwent an endovascular embolization of occipital bAVMs [64]. Although hemorrhage control is the primary objective, it is paramount to maximize visual preservation in occipital bAVMs [64]. Tawk et al. recommended the use of an amobarbital superselective injection prior to the embolization of occipital lobe arteriovenous malformations. Out of 13 patients, none developed a visual field deficit after embolization [65].

### 3.5. Treatment

Due to the vivid technological advances in the last two decades, therapeutic options for patients with bAVMs have significantly broadened and now provide a choice between microsurgery, endovascular embolization, stereotactic radiosurgery (alone or combined), and conservative treatment [66]. Interventional methods aim to completely eradicate the risk of intracerebral hemorrhages and preserve the patient’s functional status [67]. Given that subtotal obliteration of a bAVM does not fully prevent future hemorrhages, complete nidal obliteration is the main objective of definitive treatment [67].

There are numerous classification scales to prognosticate outcomes after bAVM surgery, such as the Spetzler–Martin (SM) grading scale, the simplified three-tier Spetzler–Ponce classification system, and the supplementary grading scale that complements the SM grading scale [9,68,69,70]. Likewise, the modified radiosurgery-based AVM score (RBAS) and the Virginia Radiosurgery AVM Scale (VRAS) are routinely used to predict outcomes after bAVM radiosurgery [71,72]. Recently, Meng et al. proposed using the RBAS to predict the obliteration of bAVMs after the combined use of preradiosurgical embolization and treatment with gamma-knife surgery. However, there is still no universally accepted grading scale for the endovascular embolization of bAVMs [73]. This is because all of the proposed scales for endovascular embolization, such as the Buffalo score, AVM neuroendovascular grade, or AVM embocure score (AVMES), often fail to correlate with complications (Table 1) [9,74,75,76,77].

Generally, intervention is indicated when the estimated cumulative lifetime hemorrhage risk surpasses the risk of treatment [9,59,67]. The choice of the therapeutic modality should be made by a multidisciplinary team and guided by the patient’s manifested bAVM location and angioarchitecture as well as the needs, expectations, and personal choice of the patient [59]. As shown by quality-of-life assessments, patients with untreated unruptured bAVMs have a significantly reduced quality of life. Most commonly, patients demonstrate decreased levels of health, anxiety, depression, pain, and discomfort [78]. For this reason, in cases of large bAVMs, which may lead to neurological symptoms and decline due to chronic venous hypertension and vascular stealing from surrounding tissues, the endovascular embolization of high-flow fistulae with nidal flow reduction might be an effective tool to improve the quality of life [67,79].

The therapeutic benefits of interventional therapy in the case of ruptured bAVMs; that is, preventing future reruptures, are clear. However, since the first publication of the highly controversial ARUBA (A Randomized Trial of Unruptured Brain AVMs) trial, which argued that medical management alone was superior to interventional therapy for the prevention of death or symptomatic stroke in patients with an unruptured brain arteriovenous malformation, it is still a matter of considerable discussion whether interventional therapy in unruptured bAVMs can provide a therapeutic benefit at a much lower risk of stroke or death than its natural history, thus proving the validity of choosing an interventional approach over the medical management [7,8,9]. In 2020, the ARUBA investigators published their final results after the extended follow-up, which upheld their initial conclusions [80]. Nevertheless, a significant part of the cerebrovascular community has highlighted the inadequacies of the ARUBA trial, such as the heterogeneity of patients (bAVMs with various grades), inappropriate primary and secondary endpoints, lack of standardization of the treatment arm, a design and primary hypothesis in favor of medical management, and inappropriate conclusions, among many others [81]. Similarly to the ARUBA trial, according to the observational cohort study of 204 patients conducted by the Scottish Audit of Intracranial Vascular Malformations Collaborators, conservative management compared with intervention (endovascular embolization using microsurgery, radiosurgery, or both) was associated with better clinical outcomes for up to 12 years [82]. However, the nonrandomized, observational cohort nature of this study left room for dispute. The ongoing TOBAS (NCT02098252) clinical trial should once and for all settle the debate on whether interventional therapy offers therapeutic benefits in unruptured bAVMs. In addition to evaluating whether medical management or interventional therapy will reduce the risk of death or debilitating stroke (due to hemorrhage or infarction) by an absolute magnitude of about 15% (over 10 years) for unruptured bAVMs (from 30% to 15%), this trial also aims to determine whether endovascular treatment can improve the safety and efficacy of surgery or radiation therapy by at least 10% (80% to 90%).

A large part of the literature supports the use of endovascular embolization in the treatment of bAVMs [67,83,84,85,86,87,88,89,90,91,92,93,94,95]. However, given that each neurointerventional center has a different level of expertise, there is a significant discrepancy among the reported outcomes [84,85].

This part of the review will discuss the technical background of endovascular treatment for bAVMs, different applications of endovascular embolization in the management of bAVMs, and the transvenous approach. Table 2 summarizes the chosen papers that reported outcomes when using endovascular embolization in bAVMs [67,83,86,87,88,89,90,91,92,93,94,95]. The current clinical trials of endovascular treatment for bAVMs are summarized in Table 3.

### 3.6. Technical Background

Endovascular treatment of bAVMs began with the application of n-butyl cyanoacrylate glue (nBCA) and flow-directed microcatheters to embolize the nidus [98,99,100]. However, with time, various other embolic materials have been introduced to embolize the upstream or within the arteriovenous shunt, thereby normalizing the venous pressure [98,99,100]. Liquid embolic agents (LEAs) are most commonly used; thanks to their prolonged polymerization times, they allow for a homogenous filling of the vascular area, thus limiting the risk of the secondary reopening of the embolized area [98,99,100]. LEAs can be divided into two groups: cyanoacrylates or adhesive embolic agents, which have a glue-like nature; and copolymers, which are also known as nonadhesive embolic agents. The latter have lava-like or rubber-like characteristics [98,99,100]. Among the LEAs used, we distinguished N-butyl cyanoacrylate glues (e.g., Histoacryl, TruFill^®^); ethylene–vinyl alcohol copolymer (EVOH), also known as the Onyx^®^ liquid embolic system (Micro Therapeutics, Inc., Irvine, CA, USA); SQUID (Balt, Montmorency, France); and last but not least precipitating hydrophobic injectable liquid (PHIL©; Microvention, Tustin, CA, USA) [98,99,100]. Contemporary embolizations of bAVMs rely on nonadhesive copolymers such as Onyx and PHIL due to their lower risk of catheter entrapment and longer polymerization time, which facilitate controlled injections with deeper nidal penetration [98,99,100]. Eudragit-E, a nonadhesive liquid embolic material, is not popular in use, but one study attested to its safety and effectiveness; the authors reported an overall obliteration rate of 72.7% after endovascular embolization with/without subsequent stereotactic radiosurgery [101].

Although rarely employed to occlude bAVM feeders, platinum coils may be used to effectively slow the flow in the given compartment, thus facilitating the following embolization with a liquid agent [102]. Once LEAs were introduced into the practice, the use of polyvinyl alcohol (PVA) particles was abandoned due to high recurrence rates [99].

The last decade has witnessed substantial advances in catheter tech that have significantly improved the outcomes after the endovascular embolization of bAVMs. The problem of morbidity associated with distal catheter entrapment was solved thanks to the development of detachable tip microcatheters, which have greatly facilitated the removal of the catheter from hardened liquid embolic casts [98,99,100]. The introduction of balloon microcatheters enhanced the protection of normal brain vasculature and also improved the penetration of liquid embolics into large bAVMs, which led to a reduction in the procedure times and radiation exposure [98,99,100]. Last but not least, the Scepter Mini, a recently introduced dual-lumen balloon microcatheter, allows for a more efficient and controlled injection of liquid embolic agents in smaller, more distal arteries (>1.7 mm), thereby reducing the risk of reflux [103].

### 3.7. Endovascular Techniques

**Figure 2:**
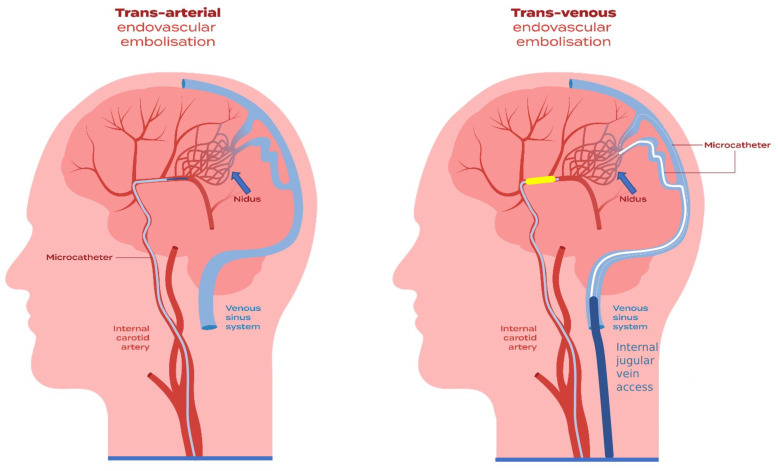
Endovascular embolization can be conducted with the use of two approaches—the transarterial approach (TAE) and the transvenous approach (TVE). A TAE can be used to support a TVE during the same or one previous preparatory session, or a TAE can be employed to rescue an incomplete TVE. In some cases, balloon catheterization can be used transarterially to assist a TVE.

To reduce the risk of catheter entrapment by Onyx reflux, Durst et al. proposed the “reverse plug then push” technique, which allows for a more swift injection of Onyx thanks to the formation of a well-controlled plug prior to treatment. The authors obtained a complete angiographic obliteration in 83% of patients after a single treatment. The average bAVM volume was 14.9 mL with a median volume of 5.85 mL and a lower and upper quartile of 0.94 and 12.5 mL, respectively. Of the bAVMs, 50% had deep venous drainage whereas 75% involved eloquent portions of the cortex. The morbidity and mortality were each 8% [104]. Traditionally, the plug-and-push technique has been successfully employed to obliterate the nidus. This technique involves the formation of a plug around the tip of the catheter to establish and increase a pressure gradient to the distal part within the nidus, thus stimulating the forward penetration of the embolization agent [105,106]. Nonetheless, due to the difficulty of controlling the level of reflux and the possible risk of occluding nontarget arteries, Chapot’s transarterial pressure cooker technique (PCT), transarterial balloon-assisted embolization, and transvenous embolization (Figure 2) have been proposed as alternatives to successfully reduce copolymer reflux and mitigate the risk of ischemic stroke or hemorrhages [105,106,107,108]. Likewise, Cekrige et al. recommended their multiplug flow-control technique to effectively control or arrest the flow during injection of a liquid embolic agent. Forming multiple plugs from microcatheters that are placed in all or multiple feeders improves the penetration of the embolic agent, which leads to shorter injection time and, thanks to flow control, the reduced washout of a bAVM. Similar to the pressure cooker technique, the multiplug flow-control technique could be potentially curative. However, its safety and efficacy require further evaluation in a larger patient group [109].

### 3.8. Endovascular Embolization in Different Clinical Settings

#### 3.8.1. Presurgical and Preradiosurgical Endovascular Embolization

Presurgical embolization of the arteries at the bAVM margins and in deep compartments of the chosen surgical exposure with partial embolization of the nidus is used to reduce intraoperative bleeding, thereby facilitating a safe and effective resection and mitigating the risk of a normal perfusion pressure breakthrough postoperatively [79]. The decision to perform preoperative embolization is strongly dependent on the location, size, and complexity of the bAVM angioarchitecture [79]. A recent report by the Society of NeuroInterventional Surgery Standards recommended preoperative embolization and stated that embolization of deep arterial feeders or those feeders that are most demanding to access surgically may be more beneficial than targeting feeders that can be easily accessed surgically [79]. Nevertheless, according to a 2022 systematic review, the evidence supporting the use of presurgical embolization was vague [86]. Luzzi et al. recognized the benefits of intraoperative hemostasis and simplified the identification of the target lesion when performing a preoperative embolization on an average of 3.7 days before resection of 27 SM grade 3 bAVMs [110], whereas a large series involving 319 patients reported no relevant differences in terms of blood loss or longer operating time with a preoperative embolization [111]. Likewise, the routine use of a preradiosurgical embolization to make bAVM more responsive to radiosurgical treatment by reducing the nidus size to <3 cm has been challenged [79,112]. Some authors supported the belief that embolization is crucial to obtain satisfactory radiosurgery outcomes and should be done in cases that allow for effective embolization; that is, not in patients with large, diffuse bAVMs for which a high-quality embolization would be extremely difficult, if not impossible [113,114,115]. However, a systematic review involving 2591 patients found that preradiosurgical embolization did not decrease the postcombined treatment hemorrhage rate and was associated with substantially lower obliteration rates than those treated with SRS alone [112]. Explanations of these results included: the embolic agent masking the nidus margin, thereby leading to a targeting error; a fragment of the nidus vanishing right after the proximal feeder occlusion because the temporary flow regression was outside of the radiosurgery target and may have later recanalized as a result of hemodynamic remodeling; and last but not least, the fact that bAVMs treated with the combined use of embolization and SRS had a more complex angioarchitecture (that is, more feeding arteries and draining veins, making radiosurgical and endovascular treatment much more demanding) [9,79,113,115,116,117]. Contrariwise, a recent multicenter-matched cohort study compared the outcomes for bAVMs that underwent stereotactic radiosurgery alone versus those with a prior embolization and found no overall differences in the obliteration rates [118]. Furthermore, upfront embolization did not have a negative impact on radiosurgery outcomes with respect to obliteration, post-SRS hemorrhages, and the overall complication profile [118]. Due to both supporting and refuting research, it remains ambiguous whether adjunctive embolization prior to radiosurgical treatment may lead to higher rates of bAVM recurrence [112,113,114,115,116,117,118,119]. If the occlusion rates are similar, the additional risk of preradiosurgical embolization might outweigh the benefits except for selective embolization of high-risk angiographic features such as intranidal or prenidal arterial aneurysms and intranidal arteriovenous fistulas to decrease the risk of bAVM rupture during the latency period between SRS and obliteration. [9,120].

#### 3.8.2. Acute Endovascular Embolization

Acute endovascular embolization involves occluding bleeding bAVM vessels to prevent fatal complications and rebleeding in the longer perspective.

Endovascular embolization is being increasingly used to selectively treat high-risk angiographic and structural components of bAVMs, especially in cases of bAVMs that cannot be resected without significant morbidity [79,121,122,123,124]. Aneurysms are very commonly associated with bAVMs. Bendjilali et al. [125], Meisel et al. [126], and Turjman et al. [127] reported that 36%, 46%, and 58% of bAVM patients, respectively, had an associated aneurysm. Once recognized as the likely source of bleeding, in particular when correlated with the pattern of hemorrhage on cross-sectional imaging, intranidal or flow-related aneurysms can be safely and effectively occluded, which substantially lowers the immediate risk of rebleeding without increasing complications [79,121,122,123,124]. Similarly, bleeding can result from venous hypertension caused by outflow stenosis, prompting specialists to reduce the arteriovenous shunting through the lesion via partial transarterial nidal embolization [79]. As pointed out in the report by the Society of NeuroInterventional Surgery Standards and Guidelines Committee, nidal compression or another form of angioarchitecture distortion caused by the mass effect of an adjacent hematoma may potentially result in the misjudgment of the actual nidus size and morphology. Consequently, curative endovascular embolization in acute clinical scenarios holds a risk of delayed recurrence [79].

#### 3.8.3. Curative Endovascular Embolization

Given the fact that reported complete obliteration rates after bAVM embolization alone rarely surpass 51%, standalone endovascular embolization has been mostly employed as an adjacent treatment method to microsurgery or radiosurgery [8,79,120,128]. However, endovascular embolization can be curative in many bAVMs, especially in cases with a small or medium volume, compact niduses, and simple angioarchitecture; that is, bAVMs supplied and drained by arteries and veins from a single vascular area [58,90,91].

Interestingly, as shown by Crowley et al. in their large case series involving 342 bAVM patients treated with endovascular embolization (median Spetzler–Martin grade III), the Spetzler–Martin grade was not associated with differences in outcome; permanent neurological deficits were only observed in 12%, 9%, 13%, 11%, and 13% of bAVMs for Spetzler–Martin grades I–V, respectively [129].

The results of studies that evaluated the efficacy of endovascular embolization in low-grade bAVMs tended to vary, thereby showing how the outcome was strongly dependent on the quality of the endovascular embolization. Pierot et al. reported 100% occlusion in 23/61 patients with bAVMs < 3 cm and 4/53 patients with bAVMs ≥ 3 cm [94]; Baharvahdat et al. obtained total occlusion in 92% of patients with low-grade bAVMs (Spetzler–Martin I–II) [87]; and Iosif et al. achieved a total occlusion of the nidus in all but one case, with 90.5% of the patients being independent in their everyday lives (mRS score 0–2) [92]. Even in cases of more complex bAVMs (Spetzler–Martin III), Baharvahdat et al. achieved total occlusion in 87.7% of patients and reported a 3% mortality rate and permanent neurological deficits in 6.2% of patients [88]. Conversely, according to a systematic review involving 597 patients with 598 bAVMs (grade ≤ III), complete obliteration immediately after embolization was reported in 58.3% of bAVMs that had complete treatment and in 45.8% of bAVMs in the entire patient cohort [130]. As a consequence, it is still under considerable debate whether it is time for a paradigm shift by using endovascular embolization to cure bAVMs rather than solely treating it as a support to microsurgery/radiosurgery or part of palliative treatment.

### 3.9. Transvenous Approach

Although the idea of venous side occlusion for bAVM treatment has been around for more than two decades, thanks to the technological advances in endovascular techniques, as well as the continuing perfection of the superselective catheterization of intracranial veins, only now has it become possible to consider the transvenous approach in previously untreatable cases; that is, bAVMs with eloquent locations, deep venous drainage, and a narrow tortuous artery supply [131,132,133,134]. Brain arteriovenous malformations with a deep venous system provide the easiest access for a transvenous approach [128]. According to Iosif, the transvenous approach offers protection from rebleeding and good clinical outcomes for patients with an otherwise poor clinical perspective without an increased hemorrhagic risk over intra-arterial techniques, granted that an appropriate injection rate is maintained in order to exclude the nidus before occluding the initial part of the main draining venous outlet [133]. These bold claims are backed by tangible results, given that the authors managed to achieve complete occlusion in 19/20 of patients with highly complex bAVMs: the initial Spetzler–Martin grades were III–V for 90.0% of the patients, and the lesions were deeply seated in 80% and in eloquent locations in 90% of the cases [133]. The safety and effectiveness of the transvenous approach were further validated by Mendes et al., who achieved anatomic obliteration in 95% of cases with a 5% morbidity rate [135]. Last but not least, in 2021, Koyanagi et al. described the transvenous retrograde pressure cooker technique (RPCT) and reported a 96% cure rate and no procedure-related mortality in 51 patients with high-grade bAVMs (SM grade III–V in 71%) that were deeply located in 30 and cortical in 21 patients (41%) [128]. Their considerable experience in the use of the transvenous approach led the authors to describe key points that should guide neurointerventionalists when considering the transvenous approach [128]. Considering that large bAVMs have large outflow veins that often are easier to navigate but are more demanding to occlude without refluxing excessively, RPCT with the intention to cure should be performed only after having obtained maximal transarterial downstaging in order to progressively reduce the caliber of the main outflow vein [128]. Secondly, in bAVMs with multiple main outflow veins, the authors recommended restricting the transvenous approach to bAVMs with a single main outflow vein except if distinct compartments with separate feeders and a dedicated draining vein can be identified and effectively embolized [128]. Thirdly, the bAVM diameter should not exceed 3 cm [128]. Last but not least, it was recommended to conduct a postembolization flat-panel CT to swiftly identify and treat perioperative rupture [128].

Although the transvenous approach provides advantages such as a decreased risk of ischemia as well as better infiltration of the embolic agent into the nidus, the effective injection of the embolic agent via the transvenous approach might be demanding, particularly in the case of high-flow bAVMs [135]. For this reason, temporary flow arrest by either balloon occlusion or adenosine-induced circulatory arrest could facilitate and improve embolization [135]. Ghorbani et al. tested the safety and efficacy of adenosine-induced circulatory arrest in the transvenous embolization of six patients with bAVMs and reported that at the 6-month follow-up, four patients had an mRS of 2 and two patients had an mRS of 1 [136]. Recently, Iosif et al. described a novel approach to their previously reported transvenous method [137] that involved placing hypercompliant balloons intra-arterially for the selective occlusion of arterial feeders during ethylene vinyl copolymer (EVOH) transvenous injection [137]. The resulting decrease in the intra-nidal pressure led to higher nidal occlusion rates: a total occlusion of the nidus was obtained in >90% of the cases at the end of the procedure and angiographic stability was obtained in all of the cases. At follow-up, 100% of the patients had an angiographic cure. Among 22 patients, 86.4% had high Spetzler–Martin’s grades [137]. After performing an MRI scan within the first postoperative month, the authors reported a 0% procedure-related mortality and a 4.5% clinically significant procedure-related morbidity [137]. In small groups of carefully selected patients at well-trained neurointerventional centers, the outcomes supported the notion that transvenous embolization with the intention to cure was effective and safe. However, there are no widely available results that were obtained in larger patient groups; any such reports have only been presented at conferences. As a consequence, not all neurointerventionalists have access to comprehensive data on this promising albeit still experimental treatment. The ongoing TATAM (NCT03691870) clinical trial should establish a reliable protocol that could guide the use of endovascular embolization of bAVMs.

## 4. Conclusions

Brain arteriovenous malformations are an incredibly challenging pathology. Due to the significant heterogeneity of bAVMs and the lack of objective data (attributable to the scarcity of evaluative or comparative studies), there is no consensus on the treatment of bAVMs. Although there is a clear benefit of interventional therapy to patients with ruptured bAVMs, it is still a matter of considerable debate whether intervention is superior to conservative treatment in the case of unruptured bAVMs. For this reason, a multidisciplinary team should evaluate each case independently while taking into consideration the manifestations, bAVM size, and angioarchitecture, as well as the patient’s needs and expectations, before deciding which treatment, if any, could be of therapeutic benefit to the given patient. Thanks to the refinement of endovascular techniques as well as advances in imaging and catheter technology, endovascular embolization can be employed as a single curative modality in the case of patients with bAVMs smaller than 3 cm that are located superficially in noneloquent areas and have fewer, larger, and less tortuous feeding arteries. Considering how bAVMs can substantially decrease the quality of life, partial/palliative endovascular embolization should be considered to alleviate manifestations in the case of untreatable lesions. The ambiguity concerning the use of presurgical and preradiosurgical embolizations should be resolved by future studies that clearly outline whether there are any benefits and, if so, in which clinical settings. Transvenous embolization of arteriovenous malformations is emerging as a safe and effective technique with leading centers now curing previously untreatable bAVMs. However, large-scale studies are necessary before any declaration of efficacy and safety can be made.

## Figures and Tables

**Table 1:** The proposed grading scales for endovascular embolization and their contents.

Graded Feature		Points Assigned
Buffalo score (Dumont and colleagues) [74]		
Number of article pedicles	1 or 2	1
	3 or 4	2
	5 or more	3
Diameter of arterial pedicles	Most > 1 mm	0
	Most ≤ 1 mm	1
Nidus location	Noneloquent	0
	Eloquent	1
AVM neuroendovascular grade (Feliciano and colleagues) [75]		
Number of feeding vessels	Less than 3	1
	3 or more and less than 6	2
	More than 6	3
Eloquence of adjacent areas	Noneloquent	0
	Eloquent	1
Presence of an arteriovenous fistula	No	0
	Yes	1
AVM embocure score (AVMES) (Lopes and colleagues) [76]		
Size of AVM nidus	Smaller than 3 cm	1
	Larger than 3 cm but smaller than 6 cm	2
	Larger than 6 cm	3
Number of arterial pedicles feeding AVM	1 or 3	1
	4 or 6	2
	More than 6	3
Number of draining veins	1 or 3	1
	4 or 6	2
	More than 6	3
Vascular eloquence *	Noneloquent	0
	Eloquent	1

AVM—arteriovenous malformation. * Defined as “Emergence of small and short arterial pedicles from parent vessel whose injury/occlusion would cause severe neurologic complications”.

**Table 2:** Outcomes of selected studies using endovascular embolization in the treatment of bAVMs.

Author (Year)	No. of Patients	No. of Embolizations	Agent Used	SM Grade	Mean Age (Years)	Presenting Symptoms	No. of Patients with Associated Aneurysms	Follow-Up Period	Mortality	Complications	Outcome	Obliteration Rate (%)
Saatci et al. (2011) [67]	350	607	Onyx (*n* = 308) Onyx with n-BCA (*n* = 42) patients	I (*n* = 52) II (*n* = 106) III (*n* = 99) IV (*n* = 69) V (*n* = 24)	34	Hemorrhage (*n* = 163) Seizure (*n* = 118) Headache (*n* = 27) Neurological signs (*n* = 29) Incidental (*n* = 13)	NS	Mean 47 months	1.4%	Permanent morbidity rate—7.4%	mRS score > 2 (*n* = 15)	51%
Sun et al. (2020) [83]	75	97	Onyx nBCA	I (*n* = 28) II (*n* = 31) III (*n* = 11) IV (*n* = 5) I–III (*n* = 70) IV–V (*n* = 5)	37.7 ± 15.5	Hemorrhage (*n* = 61) Nonhemorrhagic (*n* = 14)	44	Mean 73 months	2.7%	13.13%	0–3 mRS score (*n* = 61)	42.7%
Lv et al. (2010) [86]	147 *	220	Onyx (*n* = 76) nBCA (*n* = 144)	I (*n* = 5) II (*n* = 20) III (*n* = 54) IV (*n* = 44) V (*n* = 24)	27.5 ± 11.1	Hemorrhage (*n* = 69) Seizure (*n* = 43) Headache (*n* = 21) Focal neurologic deficit (*n* = 11) Incidental (*n* = 3)	7	NS	0%	4.8%	0–2 mRS score (*n* = 141) 3 mRS score (*n* = 6)	19.7%
Baharvahdat et al. (2019) [87]	224	319 sessions + 8 patients had more than 3 sessions	Onyx nBCA	I (*n* = 71) II (*n* = 153)	37.8 ± 16	Hemorrhage (*n* = 136) Seizure (*n* = 42) Incidental (*n* = 37) Other (*n* = 9)	62	Mean 9.7 ± 11.9 months	0.4%	Permanent neurological deficit in 5% of patients	mRS ≤ 2 (*n* = 179) 13 patients had a worse mRS score compared with their preoperative status	92%
Baharvahdat et al. (2020) [88]	65	102 + 6 patients had more than 3 sessions	Onyx nBCA	III	40.5 ± 14	Hemorrhage (*n* = 40) Seizure (*n* = 9) Isolated neurological deficit (*n* = 4) Incidental (*n* = 5)	37	Mean 12 months	3%	Permanent neurological deficit in 6.2% of patients	0–2 mRS score (*n* = 55) 3-5 mRS score (*n* = 10) Eight patients (12.3%) experienced worsening of mRS after embolization	87.7%
He et al. (2019) [89]	21	NS	Onyx	I (*n* = 3) II (*n* = 4) III (*n* = 11) IV (*n* = 3)	29.9	IC hematoma and IV hemorrhage (*n* = 15) IC hematoma (*n* = 6) SA hemorrhage (*n* = 1) IV hemorrhage (*n* = 1)	9	Mean 6 months	4.8%	Morbidity rate 4.8%	0–2 mRS score (*n* = 19)	76.2%
Poncyljusz et al. (2017) [90]	54	108	Onyx	I (*n* = 5) II (*n* = 19) III (*n* = 22) IV (*n* = 7) V (*n* = 1)	42.6 ± 15.4	Hemorrhage (*n* = 27) Headaches (*n* = 12) Seizures (*n* = 7) Focal neurological deficits (*n* = 2) Incidental (*n* = 6)	8	Mean 33.3 months	1.8%	Morbidity rate 5.6%	SM score: I–II (*n* = 24) III–V (*n* = 30)	46.3%
van Rooij et al. (2012) [91]	23	NS	Onyx	NS	42	AVM-related hemorrhagic stroke	9	21 months	1 patient died	None	No repeated hemorrhage during the 21 months of follow-up 3 patients were dependent in a nursing home and 19 patients were functioning independently	57%
Iosif et al. (2019) [92]	73	84	Onyx	I (*n* = 16) II (*n* = 57)	40.5 ± 17.8	Rupture (*n* = 44) Epileptic seizure (*n* = 6) Headache (*n* = 16) Neurologic deficit (*n* = 2) Incidental (*n* = 5)	14	6 months	0	Procedure-related morbidity was 2.7%	90.5% of the patients were independent in their everyday lives (mRS score 0–2)	Total occlusion of the nidus in all but one case
Pierot et al. (2009) [93]	50	149	Onyx (*n* = 116) Glue (*n* = 20) Onyx and glue (*n* = 13)	NS	34.8	Hemorrhage (*n* = 22) Seizure (*n* = 16) Headache (*n* = 6) Progressive neurological deficit (*n* = 2) Incidental (*n* = 4)	NS	1 month	2%	Morbidity 8%	Out of the 44 patients with incomplete occlusion after embolization, 37 were proposed for radiosurgery	Percentage of occlusion was 100% in four cases (8.3%), 80 to 99% in 27 cases (56.3%), 60 to 79% in 8 cases (16.7%), and less than 60% in 9 cases (18.7%)
Pierot et al. (2013) [94]	117	237	Onyx (*n* = 187) Onyx and glue (*n* = 37) Onyx and coils (*n* = 1) Glue (*n* = 12)	I (*n* = 20) II (*n* = 44) III (*n* = 28) IV (*n* = 24) V (*n* = 1)	42.6 ± 13.6	Hemorrhage (*n* = 40) Seizure (*n* = 33) Headache (*n* = 20) Progressive neurological deficit (*n* = 11) Incidental (*n* = 13)	32	NS	4.3%	Morbidity rate 5.1% Permanent deficits 6.0%	65/79 surviving patients needed complementary treatment	100% occlusion in 23/61 patients with AVMs < 3 cm and 4/53 patients with AVMs ≥ 3 cm
Katsaridis et al. (2008) [95]	101	219	Onyx	I (*n* = 7) II (*n* = 18) III (*n* = 39) IV (*n* = 33) V (*n* = 4)	38.8	Hemorrhage (*n* = 40) Seizure (*n* = 26) Headache (*n* = 17) Neurological deficit (*n* = 17) Incidental (*n* = 1)	NS	NS	3%	Morbidity rate 8%	48.5% are still undergoing the course of endovascular treatment with additional embolization sessions to be performed.	Total occlusion—(53.9%); near-total occlusion (34.6%)
Panagiotopoulos et al. (2009) [96]	82	119	Onyx	I-II (*n* = 59) III (*n* = 16) IV–V (*n* = 7)	44.2	IC hemorrhage (*n* = 37) Seizures (*n* = 18) Neurologic deficits (*n* = 8) Headaches (*n* = 9) Incidental symptoms (*n* = 10)	NS	6 months	2.4%	Morbidity rate 3.8%	An average of 75% volume reduction	24.4% with an average of 75% (range: 30–100%) volume reduction
Jahan et al. (2001) [97]	23	33	Onyx	I (*n* = 2) II ( *n* = 5) III (*n* = 11) IV (*n* = 5)	40	IC hemorrhage (*n* = 6) Seizure (*n* = 9) Headache (*n* = 4) Neurological deficit (*n* = 4)	NS	NS	0%	4%	Average 63% reduction in AVM volume	NS

Onyx—ethylene-vinyl alcohol copolymer, nBCA—n-butyl-2-cyanoacrylate, SM—Spetzler–Martin, mRS—modified Rankin score, NS—not stated, IC—intracranial/intracerebral, IV—intraventricular, SA—subarachnoid. * Additional gamma-knife radiosurgery was performed for 32 patients.

**Table 3:** The ongoing as well as completed clinical trials concerning the endovascular approach in the management of bAVMs.

NCT Number	Study Type	Recruitment Status	Follow-Up	Estimated Enrollment	Intervention	Objective
NCT02180958	Observational	Completed	36 months	140	Endovascular embolization	To assess the safety and efficacy of Onyx treatment for cAVM
NCT02602990	Observational	Completed	6 months	50	Endovascular embolization	To assess the safety and efficacy of SQUID™ liquid embolic agent
NCT04136860	Observational	Recruiting	5 years	1000	Conservative, microsurgical resection, embolization, embolization + radiosurgery, single-stage hybrid surgery (embolization–resection)	To assess the neurological function prognosis, occlusion rate, and complications
NCT02098252	Interventional	Recruiting	10 years	1000	Procedure: neurosurgery Radiation: radiation therapy Procedure: embolization	To assess whether: - medical management or interventional therapy will reduce the risk of death or debilitating stroke by an absolute magnitude of about 15% (over 10 years) for unruptured AVMs (from 30% to 15%) - endovascular treatment can improve the safety and efficacy of surgery or radiation therapy by at least 10% (80% to 90%)
NCT03691870	Interventional	Recruiting	3 (+/−1) months post-treatment	76	Transarterial embolization (TAE) Transvenous embolization (TVE)	The experimental treatment is an attempt to completely occlude arteriovenous malformations using venous catheterization and retrograde ethyl vinyl alcohol (EVOH) injection during the final session
NCT03209804	Interventional	Completed	12 months	519	Unsimultaneous endovascular interventional/radiotherapy followed by microsurgical resection Experimental: simultaneous endovascular embolization with microsurgical resection in a one-stage procedure	To assess the clinical benefits and risks of hybrid operating techniques in the management of cerebral arteriovenous malformations (AVMs)
NCT00389181	Interventional	Completed	5 years	226	Comparator: any combination of surgery, endovascular embolization, or radiotherapyExperimental: medical management	To determine whether medical management is better than invasive therapy in improving the long-term outcome of patients with unruptured brain arteriovenous malformations
NCT03774017	Interventional	Unknown	12 months	1200	Experimental: one-stage hybrid operationComparator: traditional microsurgical operation	To validate the benefits and risks of a one-stage hybrid operation in the treatment of complex bAVMs
NCT03031873	Interventional	Recruiting	N/A	12	MRI perfusion imaging to evaluate the evolution of progressive obliteration of the AVM nidus	Evaluation of susceptibility-weighted MRI and 4D-time-resolved magnetic resonance angiography in bAVMs

N/A—not applicable.

## Data Availability

All data gathered for the review were gathered from the articles cited in the paper and listed in the reference section.
